# Label-free optical microscopy with artificial intelligence: a new paradigm in pathology

**DOI:** 10.1117/1.BIOS.2.2.020901

**Published:** 2025-03-31

**Authors:** Chiho Yoon, Eunwoo Park, Donggyu Kim, Byullee Park, Chulhong Kim

**Affiliations:** a Pohang University of Science and Technology (POSTECH), Graduate School of Artificial Intelligence, and Medical Device Innovation Center, Departments of Electrical Engineering, Convergence IT Engineering, Mechanical Engineering, Medical Science and Engineering, Pohang, Republic of Korea; b Sungkyunkwan University, Institute of Quantum Biophysics, Metabiohealth, and Biopharmaceutical Convergence, Department of Biophysics, Suwon, Republic of Korea; c Opticho Inc., Pohang, Republic of Korea

**Keywords:** label-free optical microscopy, artificial intelligence, pathology, deep learning

## Abstract

**Significance:**

Pathological examination is essential for diagnosing diseases in tissues such as cancer but involves labor-intensive and time-consuming processes. Label-free optical microscopy has emerged as a promising alternative that offers the ability to visualize tissue structures without the need for histochemical staining. Further, the integration of artificial intelligence (AI) into label-free microscopy has the potential to streamline the overall pathological diagnostic process.

**Aim:**

We aim to review the use of AI-assisted label-free optical microscopy in revolutionizing pathological workflows.

**Approach:**

We examine the integration of AI with label-free optical microscopy techniques and assess its overall impact on the pathological workflow. We evaluate how AI enhances each stage of label-free pathology, including specimen preparation, label-free imaging, virtual staining, and diagnostic analysis.

**Results:**

Label-free optical microscopy with AI has significantly improved the entire pathological workflow. AI assists specimen preparation with high efficiency, enhances label-free imaging with high resolution and speed, and enables cost-effective virtual staining with high throughput and automatic diagnostic analysis with high accuracy.

**Conclusions:**

AI-aided label-free optical microscopy enhances diagnostic speed, accuracy, and specimen preservation, offering a transformative approach that could redefine traditional pathology workflows and improve clinical outcomes.

Statement of Discovery This work demonstrates that the integration of AI into label-free microscopy offers significant potential to enhance the entire pathological workflow, addressing challenges in specimen preparation, imaging quality, virtual staining, and diagnostic analysis.

## 1 Introduction

Pathological examination is the most widely accepted method to identify structural and functional abnormality, particularly for cancer diagnosis.^1^ As pathology has evolved with innovations in diagnostic tools and techniques, remarkable advances have increased the speed and precision of diagnoses. Digitalization of images of tissue slides has facilitated computer-aided analyses.^2^^–^^4^ However, the conventional pathological workflow involves complex processes from specimen preparation to processing, histochemical staining, and pathological assessment. This workflow is time-consuming, requires skilled technicians, and exposes specimens to potentially damaging procedures. In intraoperative situations, surgical teams need a rapid and accurate assessment of tumor margins and resection areas, which traditionally requires tissue-section analysis and histochemical staining procedures that can delay decision-making and risk of distorting the tissue. Therefore, to facilitate the diagnostic process while maintaining or improving accuracy, alternative approaches must be developed.

Label-free optical microscopy is a promising solution, which visualizes biological tissues without histochemical staining or fluorescent markers.^5^^–^^8^ Essential anatomical information can be obtained during the sample preparation and label-free imaging stages by exploiting contrast that is provided by the differing optical properties (e.g., absorption and scattering) of tissue. Imaging modalities for this purpose include bright-field imaging,^9^ autofluorescence,^6^^,^^10^^–^^12^ optical coherence tomography (OCT),^13^^–^^15^ photoacoustic microscopy (PAM),^16^^–^^28^ stimulated Raman scattering (SRS) microscopy,^29^^–^^32^ quantitative phase (QP) imaging,^33^^–^^35^ and holotomography.^36^ These techniques offer comparable image contrast and resolution to conventional whole-slide imaging while preserving tissue integrity and significantly accelerating the workflow. As complex sample preparation and chemical staining are not required, label-free optical microscopy can rapidly and quantitatively image unlabeled tissues, which is particularly valuable for intraoperative applications. High-efficiency imaging produces reliable pathological images and facilitates surgical decision-making through objective evaluation. However, the operation of sophisticated equipment and interpretation of diagnostic information from these modalities still complicate the procedures. To solve these problems and fully exploit the potential of label-free imaging, computational solutions, including virtual staining and automated image analysis, are being developed to reduce manual workload and streamline pathological workflows.

Artificial intelligence (AI) has demonstrated remarkable capabilities in improving image processing and analysis for pathology. AI, particularly deep learning (DL), has significantly accelerated the pathological workflow by automating several critical steps. For example, DL models such as U-Net,^37^ generative adversarial networks (GAN),^38^ and ResNet,^39^ which all use convolutional neural networks (CNNs),^40^ have been successfully applied to tasks such as segmentation, reconstruction, and classification of pathological images. U-Net has shown high accuracy in medical imaging with limited data; it applies an encoder–decoder architecture with skip connections. Skip connections help preserve spatial information by directly linking corresponding layers in the encoder and decoder, and thereby enable precise segmentation of areas of interest, such as cell nuclei or targeted tissue within pathological images.^41^^–^^45^ GANs consist of two primary components: a generator and a discriminator. The generator creates realistic synthetic images, such as high-resolution reconstructions or images in different staining styles, whereas the discriminator attempts to distinguish the generated images from real images.^41^^,^^46^^–^^50^ ResNet uses residual connections to mitigate the vanishing gradient problem and extract the desired feature information from input data. ResNet can classify pathological images into different categories.^51^^–^^56^ This automation increases the speed of identification of key pathological features, including tumor boundaries, cell morphology, and disease progression. Following these advances in pathology, the fusion of label-free optical microscopy and AI algorithms has shown enormous potential to improve image quality and enable automated analysis.

**Fig. 1:**
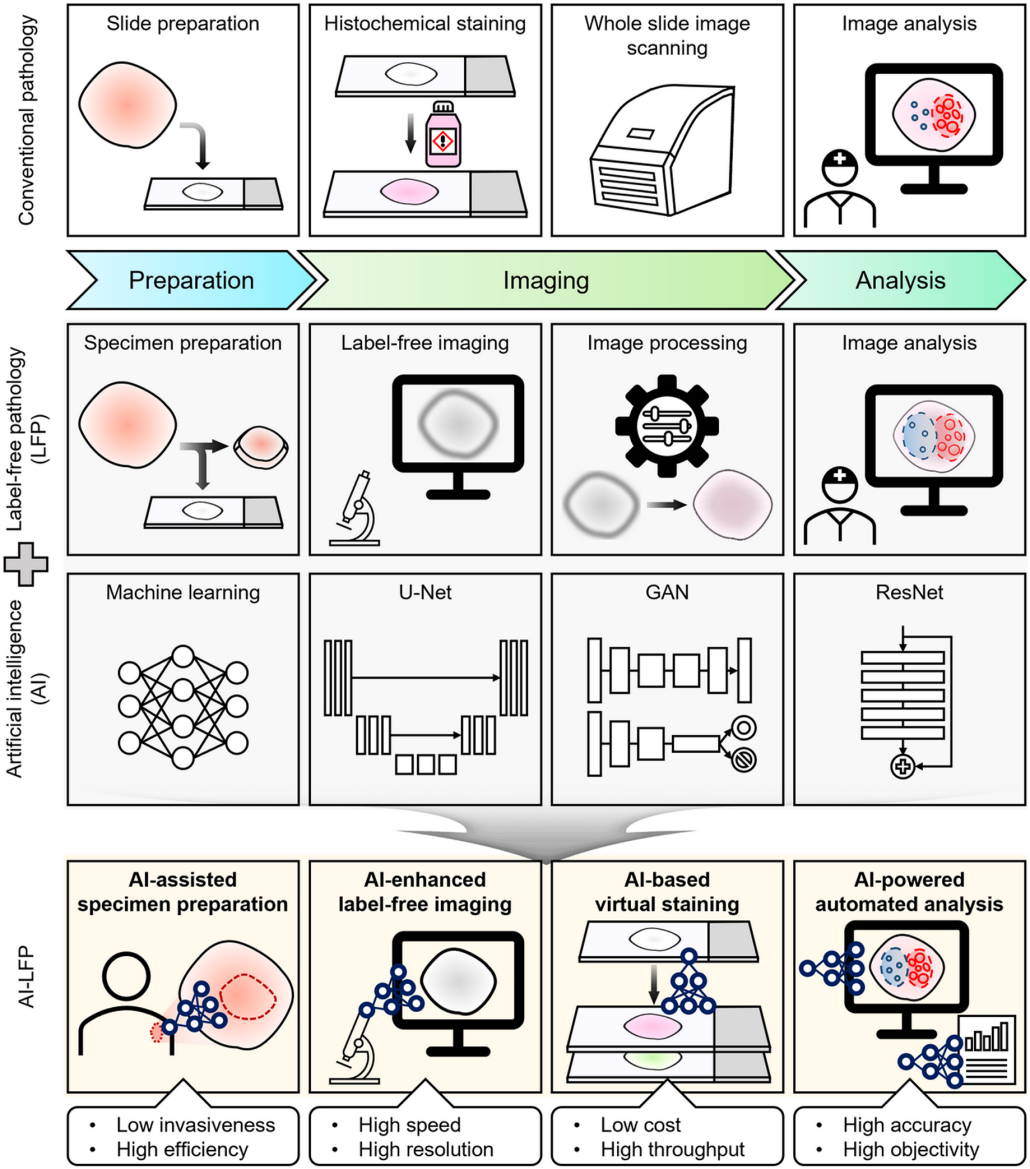
Workflow comparison between conventional pathology and label-free pathology with AI (AI-LFP), from specimen preparation to label-free imaging, virtual staining, and automated analysis.

In this review, we explore how AI-aided label-free optical microscopy is set to revolutionize pathology (Fig. 1). We examine the key stages of the pathological workflow: specimen preparation, label-free imaging, virtual staining, and analysis. At each stage, the application of AI has demonstrated the capacity to provide efficient processing and superior performance depending on its purpose. Label-free pathology with AI (AI-LFP) correctly guides efficient and minimally invasive specimen preparation, improves image quality with high-speed and high-resolution optical microscopy, facilitates diagnostic interpretation by economical and high-throughput virtual histochemical staining, and assists pathologists’ evaluations by performing objective and accurate automated analysis. Ultimately, the integration of label-free microscopy with AI in pathology offers a new paradigm, which holds the potential to redefine the traditional workflow and improve clinical outcomes.

## 2 Label-Free Pathology

**Fig. 2:**
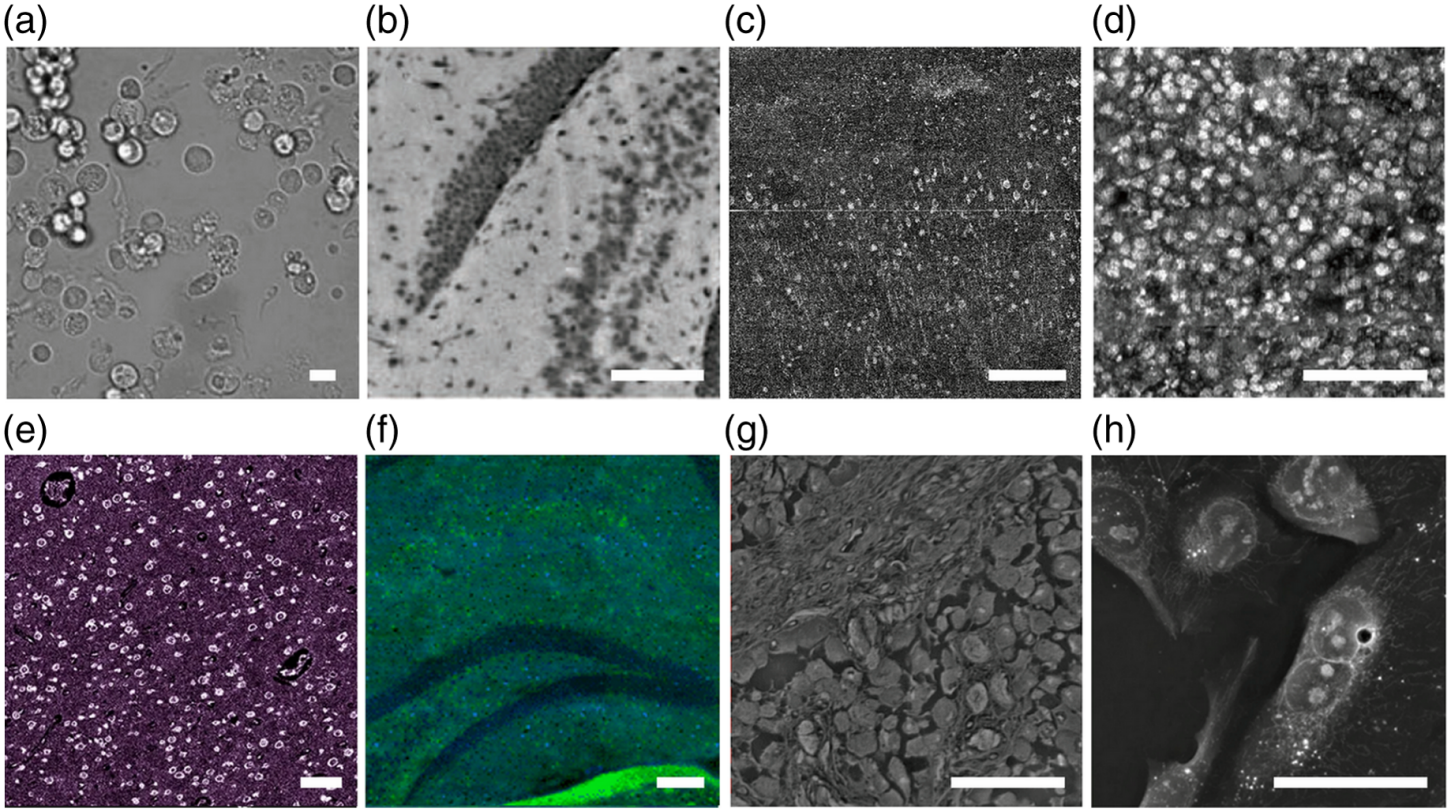
Optical microscopy technologies for label-free pathology. (a) Bright-field image of the organoid-derived cell. Reprinted with permission from Ref. 57. (b) Autofluorescence image of the brain tissue. Reprinted with permission from Ref. 58. (c) Optical coherence tomography (OCT) of the brain tissue. Reprinted with permission from Ref. 59. (d) Ultraviolet photoacoustic microscopy (UV-PAM) image of the liver tissue. Reprinted with permission from Ref. 60. (e) Ultraviolet photoacoustic remote sensing (UV-PARS) image of the brain tissue. Reprinted with permission from Ref. 61. (f) Stimulated Raman scattering (SRS) image of the brain tissue. Reprinted with permission from Ref. 62. (g) Quantitative phase imaging (QPI) of the skin tissue. Reprinted with permission from Ref. 33. (h) Holotomography image of U2OS cells. Reprinted with permission from Ref. 63. Scale bars, 100  μm.

Label-free pathology technologies offer diverse imaging capabilities by exploiting the intrinsic optical properties of biological tissues, without stains (Fig. 2, Table 1). Bright-field imaging [Fig. 2(a)], a classic approach, uses standard light absorption to capture basic tissue images, and is used as an effective preliminary examination tool in pathology. However, bright-field imaging requires thin tissue sections, and this need limits the speed of sample preparation.

**Table 1:** Comparison between optical microscopy technologies for label-free pathology. “X”: fixed and sectioned samples are required; “O”: that fresh and thick tissue samples are acceptable. Resolution is categorized as “Low” (>1  μm), “Medium” (300 nm to 1  μm), and “High” (<300  nm).

Technology	Sample preparation	Resolution	Contrast
Bright-field imaging	X	High	Absorption and refractive index
Autofluorescence	O	High	Absorption (autofluorescence)
Optical coherence tomography (OCT)	O	Low	Back-scattering
Ultraviolet photoacoustic microscopy (UV-PAM)	O	Medium	Absorption (heat)
Ultraviolet photoacoustic remote sensing (UV-PARS)	O	Medium	Absorption (heat) and scattering
Stimulated Raman scattering (SRS)	O	Medium	Raman scattering
Quantitative phase imaging (QPI)	O	High	Phase change and refractive index
Holotomography	O	High	Phase change and refractive index

By contrast, many optical microscopes can reconstruct volumetric data by comparing interactions of light and tissues and thereby facilitate sample preparation. Autofluorescence microscopy [Fig. 2(b)] exploits natural fluorescence from biomolecules and therefore can visualize morphological features.^64^^–^^68^ Autofluorescence images obtained using single- or multi-photon excitation can distinguish tissue compositions at high resolution.^69^^–^^72^ OCT [Fig. 2(c)] produces depth-resolved images by analyzing backscattered light and can achieve real-time cross-sectional imaging with minimal sample preparation.^73^^–^^75^ Along with intensity-based OCT, various types of OCT (such as full-field OCT, polarization-sensitive OCT, and spectroscopic OCT) can provide additional analytical pathological insights.^15^^,^^59^^,^^76^^,^^77^ Ultraviolet (UV)-PAM [Fig. 2(d)] provides high-sensitivity molecular contrast by leveraging the optical absorption properties of chromophores under UV laser excitation.^78^^–^^84^ UV-PARS [Fig. 2(c)], a non-contact version of UV-PAM, minimizes physical interaction with samples, so it is suitable for the analysis of delicate or difficult-to-access tissues, and therefore has utility in non-invasive biomedical imaging.^61^^,^^85^^–^^87^ SRS microscopy [Fig. 2(f)] offers high-resolution, real-time molecular imaging by detecting vibrational energy in specific chemical bonds, allowing precise biochemical profiling within tissues.^88^^–^^91^ QP imaging [Fig. 2(g)] complements these methods by quantifying differences in lengths of optical paths as light passes through tissues and thereby enables detailed cellular and microstructural analysis without staining.^92^^–^^94^ Finally, holotomography [Fig. 2(h)] applies refractive-index tomography for high-resolution 3D cellular imaging, which can provide insights into cell morphology and organelles.^95^^–^^97^

Together, label-free pathology techniques enable real-time, high-resolution visualization across various tissue types and thus offer alternatives to traditional histochemical staining methods. However, these techniques use different image characteristics, so pathologists may have difficulty understanding the images and interpreting additional diagnostic information. In the following sections, we will introduce an AI-aided label-free pathology to solve these problems and realize the full potential of label-free imaging.

## 3 AI-Assisted Specimen Preparation

**Fig. 3:**
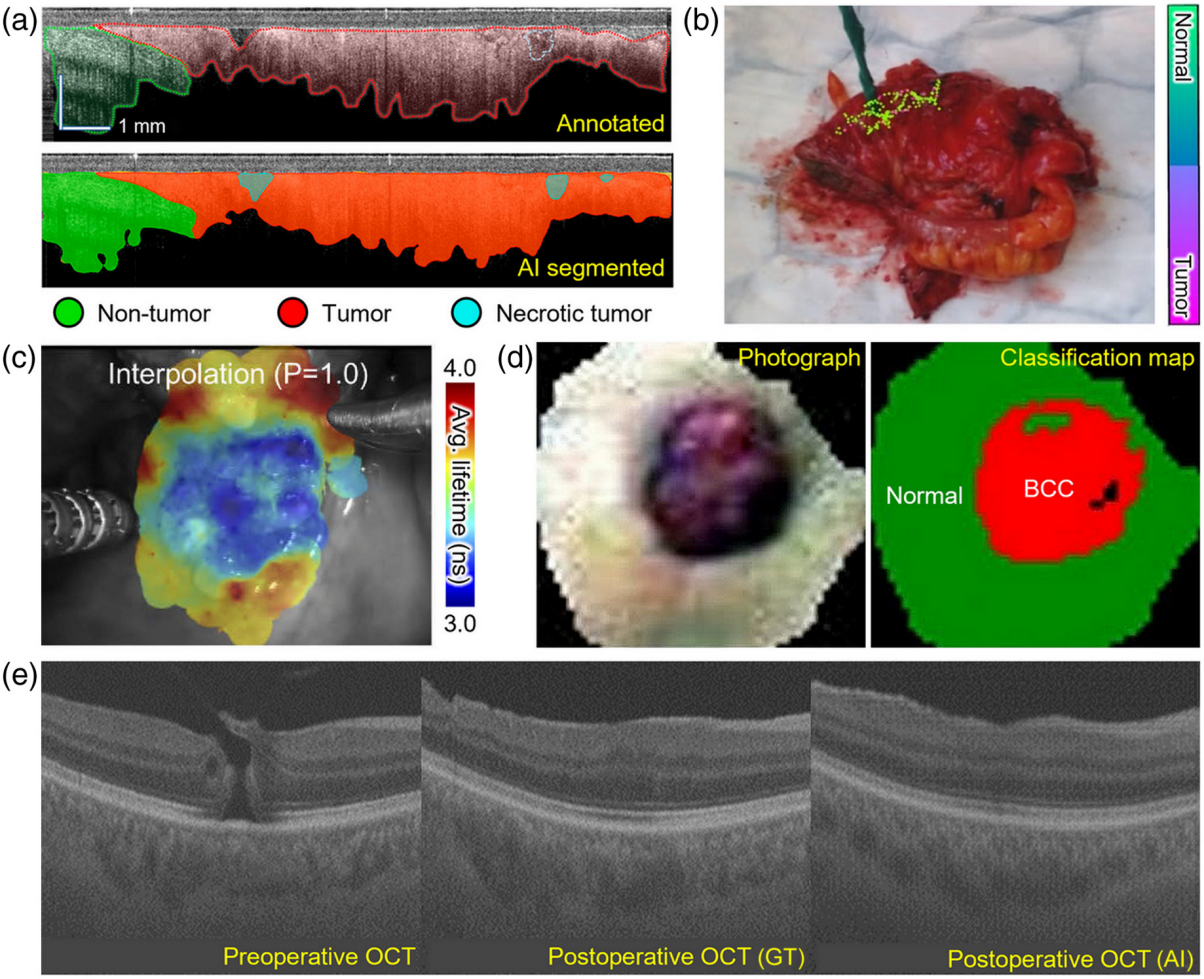
AI-assisted surgical guidance. (a) Tissue segmentation for core needle biopsy. Reprinted with permission from Ref. 98. (b) Tissue classification for tumor resection. Reprinted with permission from Ref. 99. (c) Intraoperative tumor segmentation. Reprinted with permission from Ref. 100. (d) Preoperative tumor margin classification. Reprinted with permission from Ref. 101. (e) Postoperative feature prediction for decision making. Reprinted with permission from Ref. 102.

Optical imaging is available to guide the surgical pathological workflow, including preoperative decision-making and intraoperative specimen preparation. Biopsy and surgical resection prior to pathological examination are important factors in diagnosis and prognosis. Structural boundaries and tumor margins can be identified for minimal invasion and maximal tumor resection by interpreting the anatomical information from label-free optical images. AI techniques have been implemented to improve the accuracy and reliability of tissue assessments. A core-needle biopsy guidance system that uses AI-guided OCT imaging has been developed.^98^ By training with annotated OCT images, AI-segmented and classified ones enabled highly accurate analysis of tissue compositions at the tip of the biopsy needle [Fig. 3(a)]. A CNN classifier that differentiates colorectal cancer using a handheld diffuse reflectance spectroscopy^99^ is a real-time tracking system that can visualize a probability map of cancer with high diagnostic accuracy [Fig. 3(b)]. A visualization method for intraoperative fluorescence lifetime imaging uses CNN to segment images [Fig. 3(c)];^100^ it achieves real-time interpretation with accurate localization and is therefore feasible for use as surgical guidance.^103^ Hyperspectral imaging with data classification using ML has been used to delineate the margin of a skin sarcoma [Fig. 3(d)];^101^ preoperative *in vivo* tissue assessment provided clearer boundaries than did dermoscopic images. Furthermore, to guide surgical decision-making, synthetic OCT images can predict postoperative features following surgery on macular holes^102^ and epiretinal membranes [Fig. 3(e)].^104^ Reliable simulation of surgical results can help patients and surgeons make surgical decisions. To summarize, AI can assist optical imaging by providing surgical guidance, which improves the efficiency of subsequent pathological workflows.

## 4 AI-Enhanced Label-Free Tissue Imaging

The integration of optical imaging technologies with label-free pathology enables the acquisition of high-resolution pathological images without the need for histochemical staining or sectioning and thereby shortens the overall process. However, challenges remain, including the low depth of field (DOF) of optical imaging systems, and the trade-offs between speed and resolution. Therefore, this chapter will present studies that used AI algorithms to increase the DOF of optical imaging systems while maintaining the resolution of label-free pathology and that aim to accelerate the imaging process.

### 4.1 Image Acquisition

**Fig. 4:**
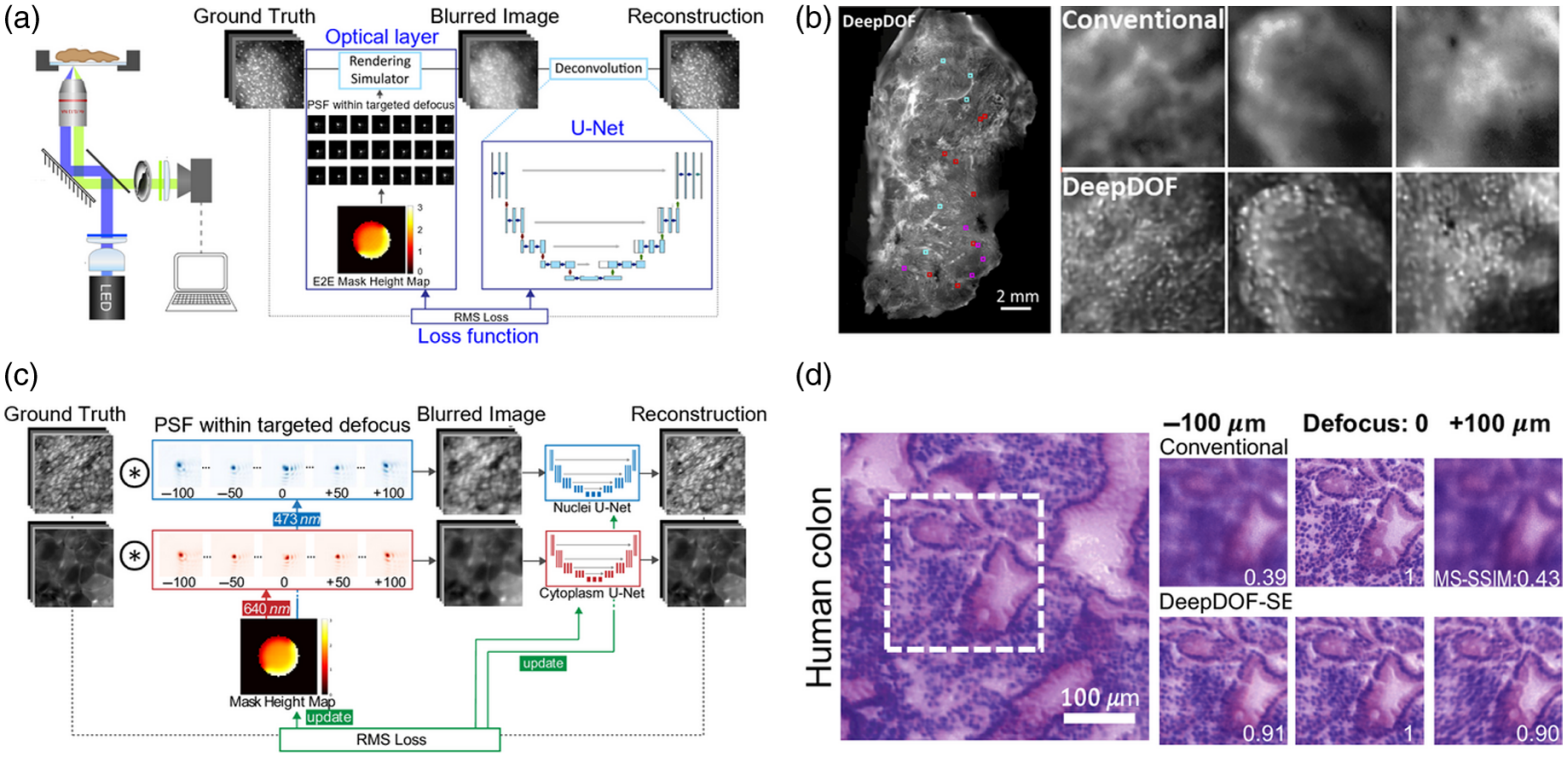
DL-extended depth-of-field (DOF) microscope for fast and slide-free pathology. (a) Schematic of the DeepDOF microscope setup with an end-to-end network for joint optimization of the phase mask and reconstruction algorithm. Reprinted with permission from Ref. 106. (b) Comparative images obtained from a large, surgically resected oral cavity specimen using the DeepDOF microscope and a conventional microscope (Olympus 4×, 0.13 NA). Reprinted with permission from Ref. 106. (c) End-to-end DL network to jointly design the imaging optics and image processing for extended DOF imaging in two fluorescence channels. Reprinted with permission from Ref. 108. (d) Images of thin (7−10  μm) human colon tissue sections acquired with DeepDOF-SE and a conventional microscope, as the sample undergoes axial translation within the target DOF. Reprinted with permission from Ref. 108.

Advances in optical imaging technologies have dramatically affected label-free pathology. Typically, high-resolution optical imaging requires a tight optical focus to achieve diffraction-limited resolution, and this tight focus restricts the DOF. This limited DOF degrades lateral resolution away from the focal plane and thereby complicates high-resolution imaging of uneven or 3D thick specimens, so the examination may require axial optical scanning and complex image processing. However, intraoperative pathology requires high-resolution imaging with an extended DOF to obtain images from uneven surfaces directly. Recently, DL techniques have been used to increase DOF.^105^^–^^108^ Deep neural networks have been trained to virtually refocus 2D fluorescence images onto specified 3D surfaces within a sample;^105^ this method, known as Deep-Z, was used to capture the neuronal activity of a *Caenorhabditis elegans* worm, using a time series of fluorescence images acquired at a single focal plane. Remarkably, this technique increased the DOF 20 times without the need for axial scanning or additional hardware and without reducing imaging resolution and speed. This approach was extended by introducing a DL-extended DOF (DeepDOF) microscope, which could rapidly image large areas of freshly resected tissue to generate histologic-quality images of surgical margins without physical sectioning while significantly extending the DOF to 200  μm [Figs. 4(a) and 4(b)].^106^ Subsequently, the same researchers presented the DeepDOF-SE microscope, which integrates the DeepDOF microscope with virtual staining of nuclear and cytoplasmic features, and thus facilitates rapid, inexpensive, and slide-free histology [Figs. 4(c) and 4(d)].^108^

### 4.2 Image Reconstruction

**Fig. 5:**
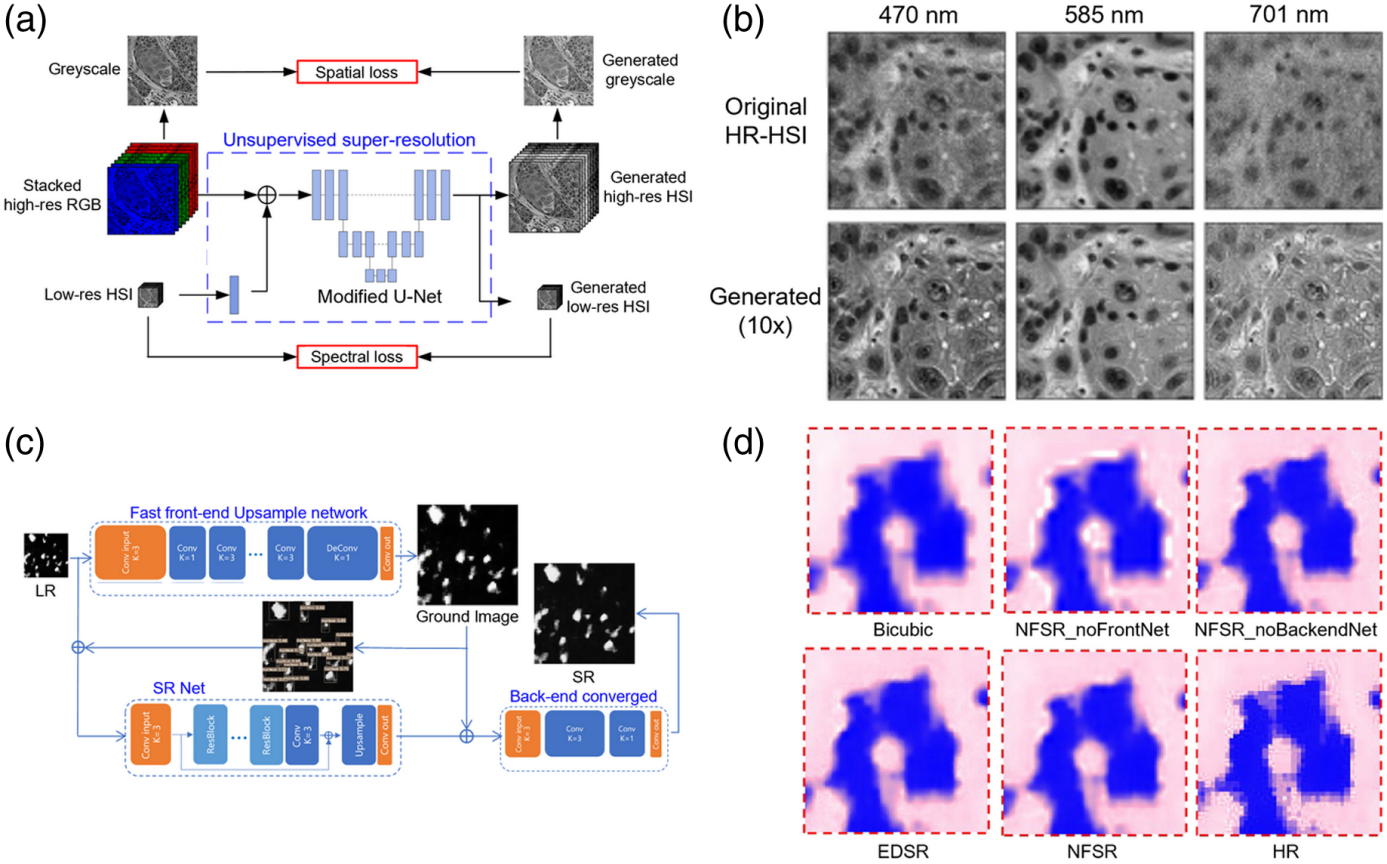
Super-resolution reconstruction of label-free pathological images using DL. (a) Architecture of the unsupervised 4× super-resolution reconstruction network. Reprinted with permission from Ref. 109. (b) Single-band images of the original high-resolution hyperspectral imaging (HR-HSI) and generated HR-HSI from three different super-resolution reconstruction networks, showing high similarity and satisfying spatial reconstruction in various wavelength bands. Reprinted with permission from Ref. 109. (c) Structure of the NFSR framework, which includes a front-end fast processing network, an object detection network, a super-resolution network, and a back-end processing network. Reprinted with permission from Ref. 110. (d) Results from the framework ablation experiment: NFSR_noFrontNet shows rendering without the front-end network, and NFSR_noBackendNet shows rendering without the back-end network, with the bicubic up-sampling method used for comparison. Reprinted with permission from Ref. 110.

Increasing the image-acquisition speed of label-free optical microscopes is crucial for advancing the clinical application of label-free pathology. However, the use of additional hardware to increase imaging speed is often hindered by high costs and complex design requirements. For example, achieving faster imaging often requires high pulse repetition rate laser sources, advanced beam-scanning systems, or high-frame-rate cameras. These components are not only expensive but also demand intricate synchronization and alignment within the optical system, significantly complicating the overall design and increasing maintenance requirements. These factors collectively make hardware-based approaches less practical for widespread clinical adoption, especially in resource-limited settings. To solve these problems, an RGB-guided unsupervised hyperspectral super-resolution reconstruction method has been developed; it increases image quality while preserving spectral characteristics [Figs. 5(a) and 5(b)];^109^ the network produced high-resolution hyperspectral images with improved quality while maintaining the integrity of the spectral data. A novel framework called non-uniform image reconstruction (NFSR) uses an object-detection network to transform low-sampled PA histology images to high-resolution counterparts [Figs. 5(c) and 5(d)];^110^ this approach accelerated PA histology imaging by a factor of 10. These methods have the potential to reduce image-acquisition time and conserve storage space without reducing image quality. A frequency-domain technique, F-mode PARS, can increase contrast in images of cell nuclei and cytoplasm. By applying unsupervised learning using principal component analysis of PARS data filtered by a set of filter banks, the method can distinguish signals from nuclear and cytoplasmic components. This F-mode processing achieved contrast-to-noise ratios of up to 38 dB between cell nuclei and surrounding cytoplasm; this was an improvement of 25 dB over their previous UV-PARS systems.^111^

## 5 Virtual Staining Using AI

Label-free pathology can highlight tissue and cellular structure information without the need for histochemical staining processes.^7^ However, most label-free pathological images are rendered in grayscale, which is hard to distinguish visually and are not as easy to analyze as conventional histochemical stained images, which are rendered in color. To overcome the limitations of label-free images, research is being conducted to develop virtual staining techniques that mimic histochemical staining, such as hematoxylin and eosin (H&E) staining,^112^ by virtually coloring them to facilitate visual understanding and increase the informativity of clinical diagnosis.

Recently, various image-to-image translation techniques that use GANs have been developed,^38^^,^^113^^–^^117^ and studies have used them for virtual staining of medical images. We have organized the latest virtual staining technologies into two types, according to their DL techniques: supervised [Table 2(a)] and unsupervised [Table 2(b)]. Supervised learning methods require accurate ground-truth data organized into pairs that match the input, whereas unsupervised learning methods can be trained using unpaired data that do not match the input exactly.

**Table 2:** Clinical label-free pathology virtual staining studies. (a) Supervised learning-based virtual staining studies. (b) Unsupervised learning-based virtual staining studies.

(a) Supervised learning-based virtual staining					
Ref.	Label-free input	Target stain	Organs	AI model	Registration
118	Autofluorescence	H&E, Jones silver, Masson’s trichrome	Salivary gland, thyroid, kidney, liver, and lung	GAN	Multi-stage registration
10	Autofluorescence	H&E, Jones silver, Masson’s trichrome	Kidney	GAN	Multi-stage registration
119	Autofluorescence	H&E	Lung autopsy	GAN	Registration DL network
120	Optical coherence tomography	H&E	Lip	Conditional GAN	—
121	Optical coherence tomography	H&E	Skin	Conditional GAN	—
33	Quantitative phase imaging	H&E, Jones silver, Masson’s trichrome	Skin, kidney, and liver	GAN	Multi-stage registration
122	Total absorption PARS	H&E	Skin	Pix2Pix	Control point registration
(b) Unsupervised learning-based virtual staining					
Ref.	Label-free input	Target stain	Organs	AI model	
123	Autofluorescence	H&E	Ovary	Cycle-GAN with input buffers	
124	Autofluorescence	H&E	Colon, breast, lung	Cycle-GAN with feature vector similarity	
125	Autofluorescence	H&E	Colorectal	Cycle-GAN with saliency loss	
58	Autofluorescence	H&E	Lung	Cycle-GAN with SSIM loss	
126	Optical coherence tomography	H&E	Skin	Cycle-GAN with segmentation and feature map loss	
62	Stimulated Raman scattering	H&E	Brain, glioma	Cycle-GAN with strongly supervised substructures	
28	UV-PARS	H&E	Breast, prostate	Cycle-GAN	
127	UV-PAM	H&E	Bone	Cycle-GAN	
60	UV-PAM	H&E	Liver	Contrastive unpaired translation with saliency loss	

### 5.1 Supervised Learning

Supervised learning requires exactly matched images, the location of the label-free image and the target pathological image must be matched using a registration method.^10^^,^^33^^,^^116^^–^^122^

**Fig. 6:**
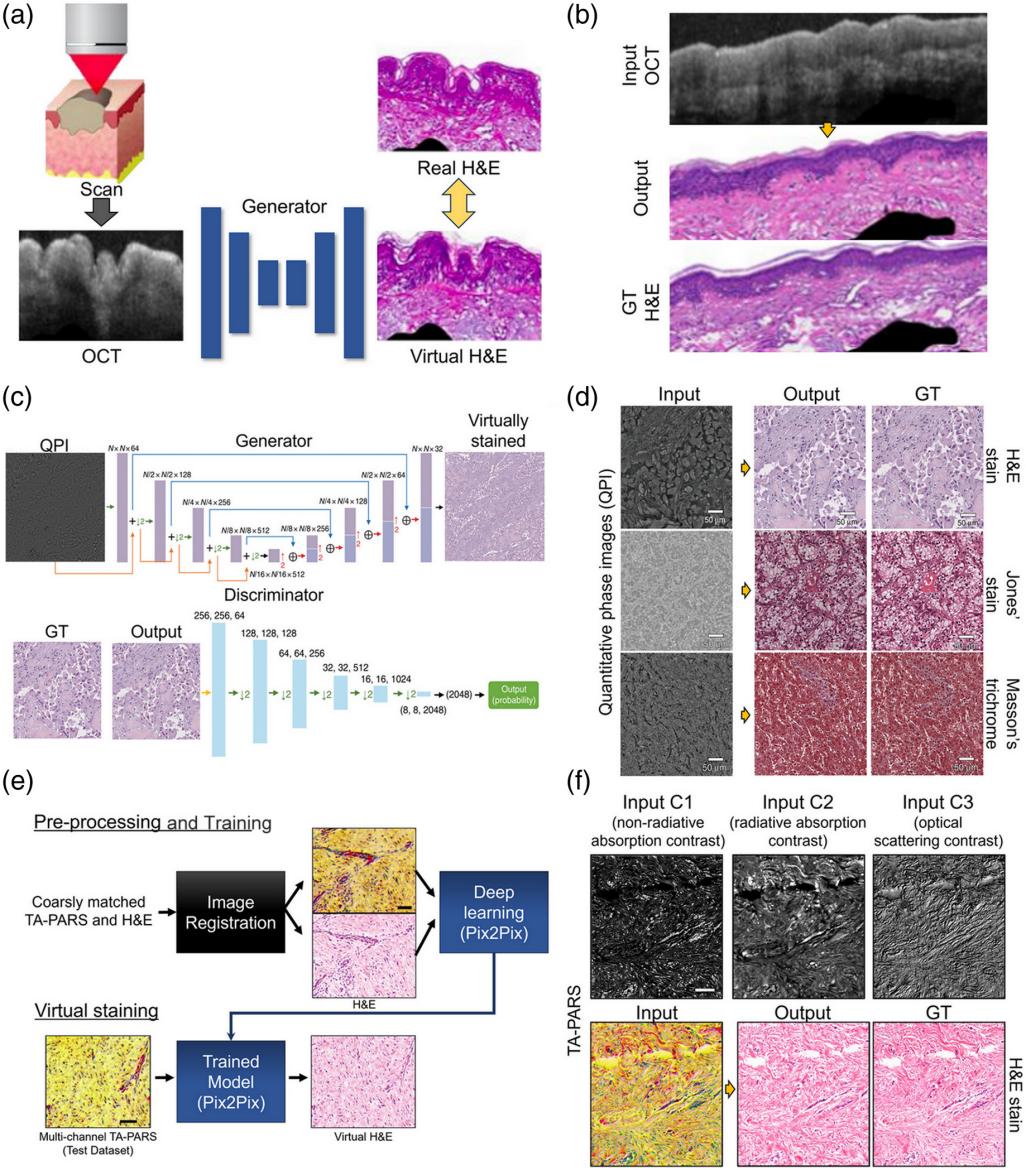
Supervised learning for virtual staining with a paired label-free image. (a) Framework for virtual staining of OCT images to virtual H&E-stained images with cGAN generator. Reprinted with permission from Ref. 121. (b) Paired OCT-H&E dataset and corresponding virtual staining results. Reprinted with permission from Ref. 121. (c) Virtual staining using a GAN model with global matching and local alignment. Reprinted with permission from Ref. 33. (d) Quantitative phase images and corresponding virtual staining results in various histochemical stains. Reprinted with permission from Ref. 33. (e) Virtual staining for H&E-style transformation from grayscale label-free TA-PARS images. Reprinted with permission from Ref. 122. (f) TA-PARS images and corresponding virtual staining results in H&E. GT, ground truth. Reprinted with permission from Ref. 122.

One proposed method virtually stained an OCT image to an H&E-style stained image [Fig. 6(a)];^121^ the researchers generated paired OCT—H&E datasets using a unique data preparation method called optical barcoding that uses fluorescent gels and showed that virtual staining using cGAN^113^^,^^115^ can be performed to obtain images similar to real H&E [Fig. 6(b)]. A virtual staining GAN model was trained using aligned QP images with multi-stage registration, including global matching and local alignment processes [Fig. 6(c)].^33^ Other methods have achieved virtual staining to match other staining targets, such as Masson’s trichrome and Jones’ stain, with great detail [Fig. 6(d)].

For grayscale, unlabeled images, which have less information than traditional, colored histochemical stained images, multiple unlabeled images can be encoded into RGB channels, and then, the results were combined to yield multichannel inputs.^28^^,^^119^^,^^122^ For example, paired training data have been constructed by registering an array of directly measured label-free contrasts such as scattering and total absorption (radiative and non-radiative) obtained from total-absorption PARS (TA-PARS) with H&E stained images, then the data have been used to perform virtual staining using the Pix2Pix^114^ model [Figs. 6(e) and 6(f)].^122^

Supervised learning virtual staining has been used in various label-free virtual staining applications due to its simple model structure and low computational cost. Also, supervised learning virtual staining can be evaluated with simple paired-image–based evaluation metrics, such as MSE, SSIM, and PSNR.^10^^,^^33^^,^^119^^,^^120^^,^^122^ However, supervised learning requires paired datasets preprocessed by the image registration process, such as multi-stage registration^10^^,^^33^^,^^118^ and DL registration methods.^119^ This difficult data preparation sequence is a serious bottleneck to real-time label-free imaging system development to adapt in clinical. Especially, in a real clincial environment, paired images of perfectly identical slices are difficult to acquire.

### 5.2 Unsupervised Learning

**Fig. 7:**
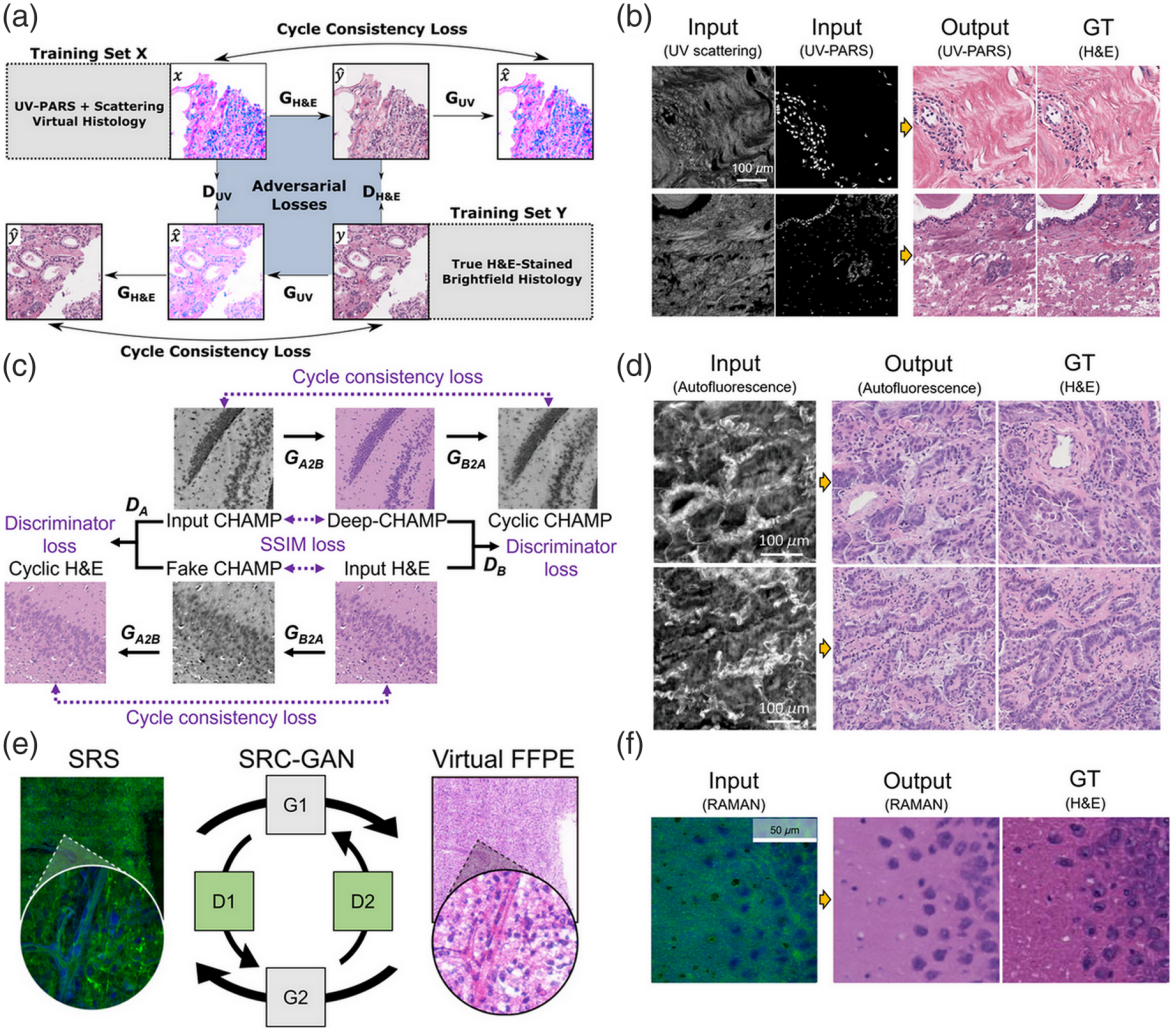
Unsupervised learning for virtual staining with unpaired label-free image. (a) Cycle-GAN framework for realistic virtual staining of UV-PARS and scattering microscopy images. Reprinted with permission from Ref. 28. (b) UV-PARS images and corresponding virtual staining results. Reprinted with permission from Ref. 28. (c) Application of SSIM loss in Cycle-GAN for virtual staining of unpaired autofluorescence images to enhance structure, contrast, and illuminance. Reprinted with permission from Ref. 58. (d) High-resolution virtual staining of human lung tissue autofluorescence images. Reprinted with permission from Ref. 58. (e) Progressive training strategy transforming paired SRS images to H&E. Reprinted with permission from Ref. 62. (f) Raman images and corresponding virtual staining results. Reprinted with permission from Ref. 62.

Image-to-image transformation techniques that use unsupervised learning have been proposed, to overcome the limitations of supervised learning.^115^^,^^117^ Starting with a cycle-consistent generative adversarial network (Cycle-GAN),^115^ which can be trained using unpaired images with cycle loss, various unsupervised learning-based image-to-image translation models have been used for label-free virtual staining.^28^^,^^58^^,^^62^^,^^123^^,^^125^^–^^130^ Cycle-GAN that applies unsupervised learning has been used to achieve realistic virtual staining on UV-PARS and scattering microscope images [Figs. 7(a) and 7(b)].^28^

Several additional techniques have been applied to existing unsupervised virtual staining methods to increase the accuracy of label-free virtual staining results. One method used Cycle-GAN that was training using the structural similarity index measure (SSIM)^131^ loss, with the goal of achieving virtual staining of unpaired autofluorescence data considering illuminance, contrast, and structure [Fig. 7(c)]^58^; and obtained high-resolution virtual staining results in autofluorescence images of human lung tissue [Fig. 7(d)]. Another method can improve results by gradually transforming the training target from paired SRS images consisting of L&P and H&E-LUTs, to unpaired H&E [Fig. 7(e)]^62^; this approach resulted in an accurate of the image transformation to be similar to real H&E images [Fig. 7(f)]. Notably, this learning strategy gradually shifts from supervised to unsupervised learning, closing the gap between paired and unpaired datasets and allowing the model to leverage the strengths of both approaches.

The advantage of unsupervised learning-based virtual staining is that it can utilize large amounts of data without additional preprocessing. However, to handle unpaired datasets, virtual staining models become more complex and need to include modules such as cyclic design or patchwise comparison,^115^^,^^117^ which inevitably increases computational cost. Furthermore, validation of virtual staining results requires evaluation methods suitable for unpaired datasets. For example, Frechet Inception Distance (FID)^132^ and Kernel Inception Distance (KID)^133^ are widely used for evaluating unpaired data, which can be utilized to quantify how similar the generated image set is to the real dataset.^60^^,^^123^ In addition, the model’s evaluation can be compared with the pathologists’ evaluation to validate the reliability of the model.^28^^,^^60^^,^^62^ However, the accuracy of unpaired data evaluation metrics is still limited because they do not target ground truth data from the exact same location, and further research is needed.

Utilizing these various virtual staining methods allows the omission of time-consuming and resource-intensive staining steps in traditional pathology workflows.

## 6 AI-Powered Automated Analysis

The ultimate goal of label-free pathology is to diagnose disease by examining tissue. Traditional manual methods for evaluating pathological images are time-consuming, and the results can vary widely among observers, so automated analysis methods that use DL are being developed to solve these problems.^134^^–^^140^ Analysis that exploits DL can maximize the efficiency of pathology analysis by understanding complex patterns and generalizing different data sets. Recently, analysis methods for label-free imaging have been developed (Table 3).^37^^,^^57^^,^^63^^,^^141^^,^^143^

**Table 3:** AI-powered automated label-free image analysis.

AI-based analysis				
Ref.	Label-free input	Analysis target	Segmentation/detection	Classification
124	Autofluorescence	Colon, breast, lung	—	ResNet-based tumor classification
125	Autofluorescence	Colorectal	U-Net–based gland segmentation	—
57	Bright-field with Fluorescence	Circulating tumor cell	Faster R-CNN–based cancer cell detection	ConvexNet-based cancer cell classification
141	Confocal	Lymph	U-Net–based cell segmentation in 2D and 3D	—
142	Deep-UV	Blood smear	U-Net based white blood cell nuclear segmentation	ResNet-based five-subtype classification (neutrophils, basophils, eosinophils, lymphocytes, monocytes)
63	Holotomography	U2OS-ACE2 cells	Two-class pixel categorization-based mitochondria segmentation and U-Net–based nuclei, nucleoli, and whole cell segmentation	—
126	Optical coherence tomography	Skin	Two CNN-based segmentation models for OCT and H&E domain	—
143	Optical diffraction tomography	Acute ischemic stroke thrombus	—	CNN-based automated prediction of thrombus composition
60	UV-PAM	Liver	U-Net-based cell segmentation	ResNet-based cancer classification

### 6.1 Segmentation

Pathology analysis methods typically use segmentation to analyze the structure of cells or tissues. This segmentation process requires labeling of the desired segmentation information. However, labeling of numerous datasets by humans is too laborious and time-consuming, so automated segmentation using DL is being researched.^37^ Especially in pathology, where high-resolution microscopy images must be analyzed, automated segmentation is an important technique.^134^^–^^137^

**Fig. 8:**
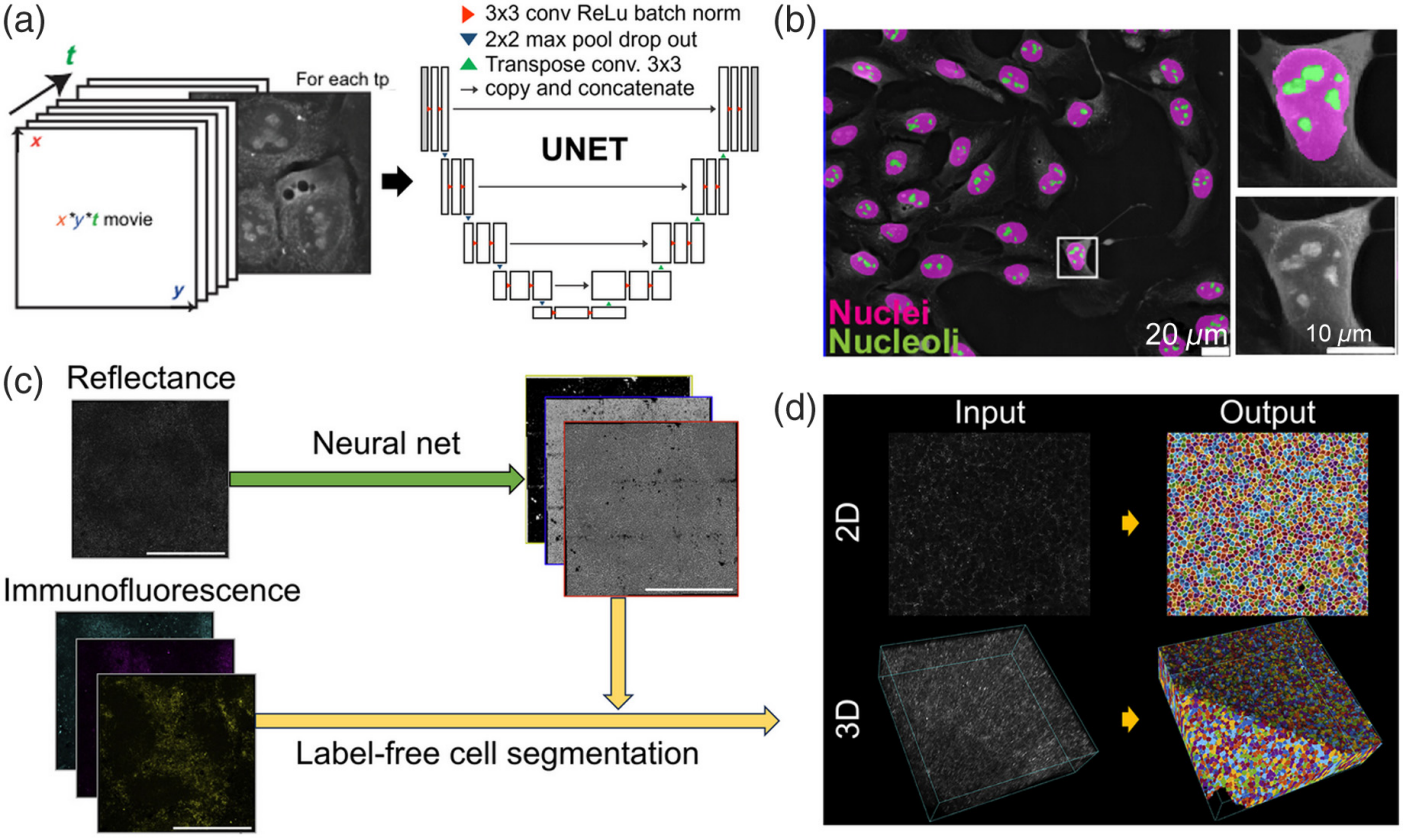
Segmentation methods for label-free pathological image analysis. (a) U-Net-based segmentation for nuclei, nucleolus, and cell detection in pathological images. Reprinted with permission from Ref. 63. (b) Visualization of intracellular changes induced by SARS-CoV-2 infection based on segmented components. Reprinted with permission from Ref. 63. (c) 2D and 3D label-free cell segmentation of cells using reflected excitation light in confocal microscopy. Reprinted with permission from Ref. 141. (d) Segmentation results with probability mapping of cellular components based on reflected light data. Reprinted with permission from Ref. 141.

Recently, segmentation methods for label-free pathology have been developed.^63^^,^^141^^,^^142^^,^^144^ The U-Net segmentation model has been used to detect nuclei, nucleoli, and cells [Fig. 8(a)]^63^ and then to compare the detected nuclei, nucleoli, and cells to visualize and quantify the intracellular changes induced by SARS-CoV-2 infection [Fig. 8(b)]. Excitation light reflected during routine confocal microscopy has been used to perform cell segmentation in 2D and 3D [Fig. 8(c)]^141^; results showed that the probability that a pixel belongs to a specified cellular component can be obtained using only reflected light [Fig. 8(d)].

By applying this segmentation process, the structural information of the target can be visualized, and additional features can be extracted to increase the amount of diagnostic information.

### 6.2 Classification

AI is also a promising technology for fast and accurate diagnosis of diseases in unlabeled images because the method can obtain diagnostic information automatically by analyzing information such as cell type, tissue pattern, presence, and severity of lesions.^137^^–^^140^^,^^145^^,^^146^

**Fig. 9:**
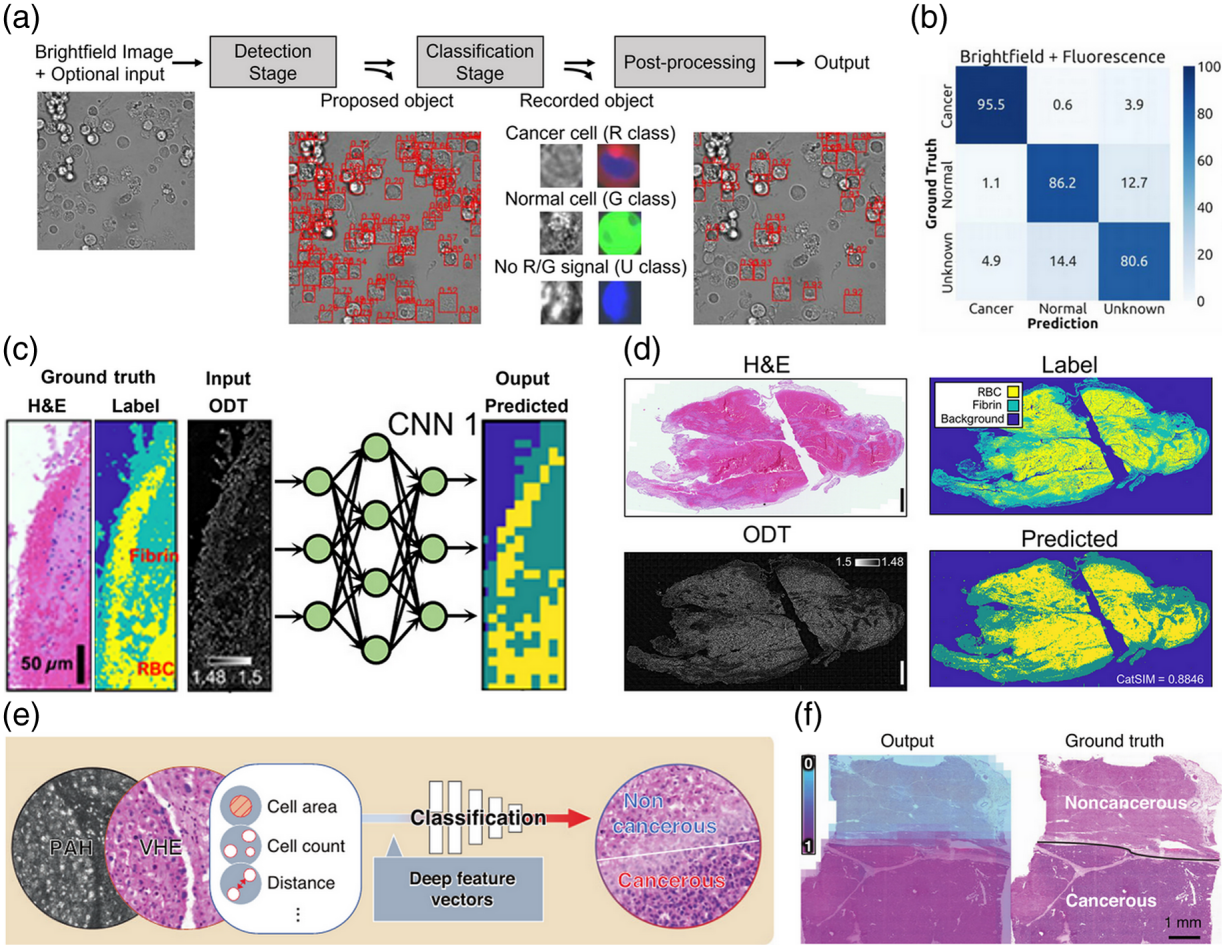
Classification methods for label-free pathological image analysis. (a) Detect and classify normal and cancerous cells in label-free bright-field and fluorescence microscopy images. Reprinted with permission from Ref. 57. (b) Classification results indicating high accuracy in distinguishing normal and cancer cells using combined bright-field and fluorescence images. Reprinted with permission from Ref. 57. (c) DL model to classify ODT image patches for automated histologic quantification. Reprinted with permission from Ref. 143. (d) Probability map showing high accuracy in distinguishing thrombus. Reprinted with permission from Ref. 143. (e) Classification framework combining UV-PAM images, virtual staining, and segmentation outputs for cancer classification. Reprinted with permission from Ref. 60. (f) Results with probability map showing high cancer classification accuracy. Reprinted with permission from Ref. 60.

DL classification using ConvexNet can distinguish between cancer cells and normal cells in label-free bright-field and fluorescence microscopy images [Fig. 9(a)].^57^ The combination of bright field and fluorescence could accurately classify cells as normal or cancerous [Fig. 9(b)]. DL has been applied to label-free optical diffraction tomography (ODT) to classify ODT image patches to permit automation of the histological quantification process [Fig. 9(c)]^143^; the method accurately classified thrombus subtypes with high accuracy without the need for staining or manual inspection [Fig. 9(d)].

By also considering the additional information (e.g., segmentation results, virtual staining results, other label-free imaging modalities) that can be obtained from label-free images, increased accuracy of clinical diagnosis can be expected. Cancer classification accuracy can be increased by combining information from UV-PAM images, additional virtual staining results, and segmentation results [Fig. 9(e)]^60^; DL analysis that used all of this information obtained excellent results that were similar to the actual classification obtained using histological images and were more accurate than results obtained using only the label-free image as input [Fig. 9(f)].

These analytical methods not only play a crucial role in label-free image analysis but also serve as key evaluation tools for virtual staining techniques, addressing the limitations of traditional virtual staining evaluation methods. For instance, cell segmentation offers quantitative insights into cellular morphology and tissue architecture, enabling a more detailed and objective assessment of virtual staining results.^58^^,^^60^^,^^123^^–^^125^ By bridging the gap between label-free pathology and conventional pathology, these methods enhance the reliability and applicability of label-free pathology.

## 7 Discussion and Conclusion

From specimen preparation to analysis, AI contributes to a revolution in the workflow of label-free pathology analysis, by overcoming the limitations of conventional label-free pathology, and by increasing the efficiency of the analysis. Table 4 tabulates the comparison between the AI-LFP and conventional pathology. AI-LFP has superiority comparable to conventional pathology in terms of time and cost efficiency and data effectiveness. The specimen preparation and staining procedures are simplified, allowing for faster examination, and AI-based analysis yields accurate and consistent results. Although initial equipment investment is required, it is efficient in the long term through cost reduction and automation.

**Table 4:** Comparison between AI-LFP and conventional pathology.

		Conventional pathology	Label-free pathology with AI (AI-LFP)
Time	Specimen preparation	Slow (>30 h, including FFPE tissue section, H&E staining)	Fast (<1 h, no staining)^28^^,^^62^^,^^127^
	Diagnostic analysis	Slow (>1 h, manual)	Fast (<10 min, automatic)^137^^,^^142^
Cost	Equipment	High (>$100 k, including whole slide scanner and microtome)	High (>$100 k, including laser, imaging system, and AI server)
	Supplies	High (>$50 k/year, including staining tools, slides, etc)	Low (<$20 k/year, including processing and maintenance)
	Labor	High (>$300 k/year, manual)	Low (<$50 k/year, automatic)
Data	Accuracy	High (manual)	High (automatic, quantitative)
	Consistency	Low (manual)	High (automatic, objective)
	Throughput	Low (manual)	High (automatic)
	Scalability	Low (individual staining and analysis)	High (label-free, virtual staining)

At the specimen-preparation stage, optical imaging integrated with AI can guide preoperative decision-making and intraoperative specimen handling. In this part, we discuss how noninvasive imaging technologies such as OCT and spectroscopy can exploit additional AI to significantly improve surgical guidance, which affects diagnosis efficiency and the patient’s prognosis. The AI-assisted specimen preparation as a basis of label-free pathology increases the quality of overall pathological workflow.

Recent advances in AI-enhanced imaging acquisition and reconstruction have significantly improved the capabilities of label-free optical microscopy and have thereby overcome several key limitations inherent in conventional methods. One of the primary challenges is limited DOF, which restricts the ability to obtain high-resolution images of thick or uneven specimens. AI-driven techniques have successfully extended the DOF without the need for complex hardware or axial scanning and have thereby enabled the imaging of fresh tissue. Moreover, reconstruction techniques that apply AI can reduce image-acquisition time while preserving high resolution. Such advances can increase both imaging speed and resolution without requiring costly hardware upgrades.

To go beyond simply improving existing label-free light microscopy images and replacing traditional pathology, label-free microscopy must address the visual limitations of mostly black-and-white information. To overcome the visual limitations of label-free imaging, a virtual staining system that applies AI has been introduced to enhance the visualization and understanding of images. We have reviewed methods that use supervised learning or unsupervised learning to provide virtual staining and have discussed their strengths and weaknesses. Supervised methods that rely on paired image datasets can achieve higher accuracy and consistent output quality because they utilize aligned input and target images, but they are less flexible and scalable because they require demanding preprocessing to obtain paired datasets. Unsupervised methods, on the other hand, utilize unpaired datasets, which provides greater flexibility. However, the lack of supervision can sometimes lead to inaccurate results, and the complex model configuration for processing unpaired images can increase computational demand. Therefore, when choosing the appropriate virtual staining model for a label-free imaging system, users must consider data characteristics, model complexity, and speed as a whole.

Finally, during the analysis stage to obtain diagnostic information, which is the goal of pathology, AI enables automated, highly accurate, and rapid analysis of high-resolution label-free microscopy images to serve as a diagnostic aid. In the case of label-free images, which contain less information than conventional pathological images and are difficult for pathologists to diagnose, such an automated analysis system can improve the accuracy of diagnosis and greatly improve the utilization of label-free images.

The combination of label-free optical microscopy and AI therefore shows promise as a new paradigm in pathology. AI-LFP has been applied over multiple pathological stages to demonstrate its clinical feasibility. For example, Jiang et al.^147^ used intraoperative label-free imaging using SRS and rapid automated AI analysis to make accurate surgical decisions in skull base tumors. Yoon et al.^60^ improved the diagnostic accuracy of hepatocellular carcinoma by interconnected feature fusion of reliably virtual stained PAM images with H&E WSIs. Ma et al.^148^ imaged cervical tissue with high-resolution 3D optical coherence microscopy and performed diagnostic classification by linking medical records with patient information via AI techniques. However, before they can be fully adopted in clinical practice, several technical challenges must be overcome. First, in terms of surgical guidance, systems for use in intraoperative applications must be fast and have high and consistent accuracy. Second, to fully realize the potential of label-free optical microscopy in intraoperative pathology, the imaging technique must be further advanced.^149^^–^^153^ Third, virtual staining results must be demonstrated to be equivalent to conventional histochemical staining. Finally, for automated analysis systems for label-free images to serve as an effective analysis assistant, the analysis accuracy and reliability of results must be increased. In clinical practice where labeling numerous data is limited, the weakly supervised learning method can be used for grading severity and predicting metastasis in tumor diagnosis by analyzing intrinsic features in various domains.^154^^,^^155^ Other challenges include the generalization of AI models to different tissues and label-free imaging techniques, the lack of a label-free data environment, and the insufficient transparency and explainability of the AI system. If these clinical hurdles can be overcome, the combination of label-free and AI could become a key technology in future pathology.

## Data Availability

Data sharing is not applicable to this article, as no new data were created or analyzed.
